# ‘It’s not just thrush, it’s recurrent thrush’: a qualitative study of patient and clinician perspectives on diagnosing recurrent vulvovaginal candidiasis

**DOI:** 10.3399/BJGP.2025.0437

**Published:** 2026-06-02

**Authors:** Tori Ford, Sue Ziebland, Sarah Tonkin-Crine, Gail Hayward, Abigail McNiven

**Affiliations:** 1 Nuffield Department of Primary Care Health Sciences, Radcliffe Primary Care Building, Radcliffe Observatory Quarter, Oxford, UK

**Keywords:** diagnosis, patient perspectives, primary health care, qualitative research

## Abstract

**Background:**

Existing qualitative research reports patient dissatisfaction with diagnostic processes for recurrent vulvovaginal thrush in primary care. This dissatisfaction centres on perceived diagnostic delays and a lack of clinical awareness of guidelines.

**Aim:**

To understand how patients and healthcare professionals experience and perceive the diagnostic process for recurrent thrush in UK primary care and what can be learned to improve diagnostic pathways, clinical encounters, and patient experiences.

**Design and setting:**

A qualitative study of patient experiences with recurrent vulvovaginal thrush and healthcare professional perspectives about providing care was undertaken.

**Method:**

Qualitative interviews were conducted with 32 people who self-identified as having recurrent thrush and 25 healthcare professionals working in primary care and sexual health services in England. Data were analysed using reflexive thematic analysis to develop themes across and within the datasets. The Candidacy Framework was then applied to interpret findings in relation to access to diagnosis and care. Patient and public involvement informed topic guide development and interpretation of results.

**Results:**

Differing expectations, perceptions, or understanding of recurrent thrush led patients to perceive missed opportunities in diagnostic pathways. The diagnostic process by which patients and primary care professionals identified recurrent thrush is presented through the candidacy framework stages of: identifying candidacy; navigating services and permeability; (re)appearing at services; adjudication; and offers and resistance.

**Conclusion:**

Diagnosis was recognised as a complex process that involved identifying patterns of recurrence, documenting this recurrence, accessing testing, interpreting test results, and forming a diagnosis as a label that opened up management pathways. Implications for practice are presented.

**How this fits in**

Patients with recurrent vulvovaginal thrush report perceived delays and dissatisfaction with diagnostic processes, often attributed to a lack of clinician awareness. Previous research highlights patient perspectives; however, few studies have included views from primary healthcare professionals. This study combines patient and clinician perspectives to explore how recurrent thrush is identified, investigated, and diagnosed in primary care. Using the Candidacy Framework, it highlights specific stages in the diagnostic process where perceived missed opportunities commonly occur. The findings highlight how differing expectations, perceptions, or understanding of recurrent thrush led patients to perceive missed opportunities in diagnostic pathways. This study suggests opportunities to strengthen care by improving communication, capturing pattern recognition, and establishing collaborative timelines.

## Introduction

Recurrent vulvovaginal candidiasis, commonly known as thrush, is defined in UK clinical guidelines as ≥4 episodes in a 12-month period with two episodes confirmed by microscopy and/or culture.^1,2^ Diagnosis typically occurs in primary care or sexual health services with symptom assessment and investigations. Acute thrush is defined as the ‘first’ or ‘single’ isolated presentation of thrush with symptoms of vulvovaginal itching, soreness, irritation, burning, and a potential change in vaginal discharge.^1,2^ Unlike acute thrush, recurrent thrush diagnosis necessitates identifying a pattern over time.

Existing qualitative research on recurrent thrush diagnostics has mainly explored symptom identification and its emotional impact, whereas the process of receiving a clinical diagnosis remains underexplored.^3–6^ Several studies have found that patients frequently bypass healthcare professionals, turning instead to self-diagnosis. This was often driven by a need for timely symptom relief.^3,7^ The few studies that explore clinical diagnosis highlight patient dissatisfaction and perceived diagnostic delays. Reported barriers include inconsistent examinations, irregular swab collection, widespread self-(mis)diagnosis, and requiring multiple visits to healthcare professionals.^8,9^ An Australian qualitative study reported diagnostic delays ranging from 9 to 48 months, with patients describing a perceived lack of clinician awareness regarding diagnostic criteria.^10^

Research into healthcare professionals’ perspectives on diagnosing and managing recurrent thrush remains limited.^8^ A focus group study with Dutch GPs found that healthcare professionals seeing patients with recurrent vulvovaginal symptoms reported frustration, discomfort, and helplessness when diagnosis was unclear.^11^ This sense of diagnostic ambiguity is compounded by the wide differential for vulvovaginal symptoms where a diagnosis is sometimes not straightforward or cannot always be reached.^12,13^ Numerous studies report that conditions like lichen sclerosus are repeatedly misdiagnosed as recurrent thrush, leading to delays in treatment, worsening symptoms, and other potential complications.^11,14,15^ There have been calls to develop better services for prompt and accurate diagnosis of recurrent and persistent vulvovaginal discomfort.^16^

More broadly, qualitative research on gendered health conditions and undifferentiated symptoms has highlighted tensions around perceived diagnostic delays. For example, a study on endometriosis found a potential mismatch between patients’ perceptions of care and GPs’ considerations, and this is not owing to GPs simply lacking awareness.^17^

Our previous work highlights the need to move away from viewing recurrent thrush as a series of minor, acute, and one-off episodes, and to recognise it as a distinct condition with unique considerations.^18^

The aim of this study was to understand how patients and healthcare professionals experience and perceive the diagnostic process for recurrent thrush in UK primary care, and what can be learned to improve diagnostic pathways, clinical encounters, and patient experiences.

## Method

A qualitative design was chosen to explore how recurrent vulvovaginal thrush is recognised and diagnosed in primary care. Narrative and semi-structured interviews were chosen as this approach enabled in-depth accounts to be captured in participants’ own terms.

A patient representative group of seven women and one non-binary person (age range: 23–80 years old) supported the design and dissemination of recruitment materials, interview guides, and initial analysis. Patient recruitment took place via posters in GP offices, sexual health centres, community pharmacies, and community centres, and through online channels such as support groups and social media. Healthcare professionals were recruited via clinical research networks, newsletters, and posters in relevant settings. We purposively sampled for maximum variation across demographics (ethnicity, gender, age) and experiences.^19^ Informed consent was obtained from all participants before interviews began.

Patient interviews began with a narrative prompt: participants were invited to describe what happened from the moment they first suspected something was wrong.^20^ This was followed by a semi-structured interview to explore key topics in more depth.^21^ Interviews were conducted via video call, phone, or in person based on participant preference.

For healthcare professionals, we used a vignette-based approach. Participants were presented with a fictional clinical scenario, developed with input from patients and clinicians, and asked to reflect on how they might respond.^22^ This method was intended to elicit detailed responses without asking to disclose information about specific patients.^17,23^ Semi-structured questions were laid out in a topic guide between layers of the vignette.

All interviews were audio-recorded, transcribed verbatim, anonymised, and analysed in NVivo 12. Analysis was conducted separately for patient and clinician data. The first author carried out interviews, reflexive thematic analysis, and initial theme generation that were then reviewed in discussion with the wider study team. Then, all codes relevant to diagnosis were exported and analysed together using the one sheet of paper method (OSOP).^21^ We then returned to the literature and used the Candidacy Framework to further inform analysis.

### The Candidacy Framework

The Candidacy Framework presents an entry point into understanding how people come to be deemed eligible for health care and are processed through systems as ‘candidates’ for care.^24^ It presents features for understanding access: identification, navigation, permeability, appearances, adjudications, offers and resistance, and operating conditions.^24^ This model has been used to explore access to general practice as a dynamic, negotiated process between patients and healthcare professionals, and has been expanded for chronic health conditions.^25,26^

## Results

Interviews were conducted with 32 patients self-identifying with recurrent thrush (May 2022 to June 2023) and 25 primary care or sexual health professionals (May to July 2024). Patient interviews lasted 45–90 min; clinician interviews 30–60 min. Pseudonyms are used throughout: patients selected first names; while healthcare professionals are labelled with pseudonym initials.

Of the patient participants, 22 reported having at least one positive swab for *Candida albicans*, two for *Candida glabrata*, two tested negative, and six said no swabs were taken. Tables 1 and 2 display participant characteristics.

**Table 1.** Characteristics of patient participants

Name	Age	Gender^a^	Ethnicity^a^	Duration (years)	Swab results
KJ	42	Gender fluid	White British	>10	Positive
Ayesha	25	Woman	Pakistani	4	Negative
Teddy	21	Non-binary	White British	3	None taken
Nancy	37	Woman	White British	6	Positive
Emily	32	Woman	White British	10, resolved	Positive
Aditi	22	Woman	Indian British	2, resolved	None taken
Sai	24	Woman	Indian	4	None taken
Ella	50	Non-binary	White British	6	Positive
Nysha	40	Woman	Black British	>10	Positive
Beth	25	Woman	White British	10	Positive
Laura	42	Woman	White British	>10	Positive
Imani	35	Woman	Black African	2, resolved	None taken
Jody	26	Woman	White British	5	Positive
Elliott	30	Trans man	White British	2	Positive
Billie	25	Woman	White British	7	Positive
Joy	43	Woman	White British	9 months	None taken
Sasha	34	Woman	Black African	1	None taken
Zoya	33	Woman	Pakistani	3	Positive
Kayla	42	Woman	White British	6	*Candida glabrata*
Lydia	26	Woman	White British	1	*Candida glabrata*
Leah	26	Woman	White British	10	Positive
Anna	34	Woman	Mixed race	10, resolved	Positive
Georgia	27	Woman	White British	4	Positive
Emma	41	Woman	White British	>10, resolved	Positive
Marie	60	Woman	White British	5	Negative
Harry	25	Woman	White British	2	Positive
Julia	36	Woman	White British	>10	Positive
Hannah	31	Woman	White British/Polish	2	Positive
Chloe	30	Woman	White British	>10	Positive
Imogen	29	Woman	White British	>10, resolved	Positive
Rowan	24	Woman	White British	2, resolved	Positive
Sarah	35	Woman	White British	10	Positive

^a^Gender and ethnicity were self-identified.

**Table 2.** Characteristics of healthcare professional participants

**Name**	**Role**	**Age**	**Gender^a^**	**Ethnicity^a^**	**Experience (years)**	**Location**
GP1 Dr A	GP partner	52	Woman	White British	25	Oxford
GP2 Dr B	Foundation year doctor	24	Woman	White British	2	Plymouth
GP6 Dr F	GP	35	Woman	White British	7	Oxford
GP8 Dr H	GP	32	Woman	British Pakistani	2	Leicester
GP9 Ms I	Physician associate	35	Woman	Indian	10	South Yorkshire
GP11 Dr K	GP	51	Woman	White British	13	Oxford, Shetland
GP13 Dr M	GP partner	33	Woman	White Irish	10	Stalybridge
GP16 Dr P	GP partner	44	Woman	White British	11	London
GP17 Dr Q	GP	53	Woman	White British	22	Clitheroe
GP18 Dr R	GP	37	Woman	White British	6	Birmingham
GP20 Dr T	GP	36	Man	White British	3	Manchester
GP21 Dr U	GP	33	Man	Black African	8	Nottingham
GP23 Dr W	GP	35	Woman	British Chinese	5	Stockport
GP24 Dr X	GP community gynaecology	40	Woman	White British	12	Oxford
SH3 Ms C	Registered nurse	31	Woman	White British	5	Manchester
SH4 Dr D	SH consultant	38	Man	White British	13	London
SH5 Dr E	SH doctor	34	Man	Asian British	4	London
SH7 Dr G	SH doctor	43	Man	Asian	15	London
SH10 Dr J	GUM consultant	59	Woman	White Other	29	London
SH12 Dr L	SH doctor	32	Woman	Black/White	3.5	London
SH14 Dr N	SH consultant	42	Woman	White Irish	15	Manchester, Glasgow
SH15 Dr O	SH consultant	47	Woman	White British	16	Devon
SH19 Dr S	SH doctor, GP registrar	29	Woman	Asian/White	2.5	Cornwall
SH22 Dr V	SH doctor	38	Woman	White British	9	Manchester
SH25 Dr Y	SH associate specialist	59	Woman	White British	15	Sussex

^a^Gender and ethnicity were self-identified. GUM = genitourinary medicine. SH = sexual health.

The journey of recurrent thrush diagnosis is presented through the Candidacy Framework. Some stages have been combined and applied to both patient and healthcare professional experiences.

### Identifying candidacy

#### Recognising recurrent thrush

Participants shared that thrush became seen as something requiring medical attention when signs and symptoms changed in duration, frequency, and/or intensity. While recurrent thrush is defined as four or more episodes within a 12-month period, patients often reported higher rates of recurrence prompting them to seek care, often on a monthly or cyclical basis. Three participants labelled their thrush experience as persistent or chronic.

Many patients shared that, early on, they assumed that their symptoms of itch, irritation, and discharge were caused by thrush and began self-treating with over-the-counter medications without first being assessed by a healthcare professional. This approach may be appropriate for patients with a one-off, acute, and transient case of thrush, but became problematic when thrush became recurrent:

*‘I did a WebMD, worked out that it was probably thrush, started taking the medication, and it went away. Then I got it every 2 months for a year and a half* […] *It was like, “I don’t think this is normal, I think this is a recurring thing.”’* (Teddy, 21 years, non-binary, patient)

When patients began to worry about their approach, consider further investigations, or seek confirmation of a diagnosis, they turned to primary health care.

However, patients reported difficulty having healthcare professionals move away from diagnosing an acute thrush episode and towards recognising and acknowledging a recurring or persistent condition:

*‘They did a swab and said, “You have thrush, here’s fluconazole.” Then I had telephone appointments, and they said, “sounds like you have thrush again”, treating it like a discrete one-off thing.’* (Rowan, 24 years, woman, patient)

There were different views about whose responsibility it was to begin conversations around recurrence. Some patients were waiting to be asked directly, and others were unsure how to begin building a diagnosis:

*‘I don’t think anyone asked me how often I had it, just people saying “Go to the doctor if you have that a lot.” I have gone, but it is treated like an acute episode.* [Nobody] *asked the pertinent questions in terms of giving me a proper diagnosis*.*’* (Sasha, 34 years, woman, patient)

Healthcare professionals explained that existing systems were not well equipped to identify and address conditions that are typically one-off, but could become recurrent or persistent:

*‘The gap is the transition between seeing thrush as a self-limiting one-off condition and then having it* [thrush] *often. For our health systems, making the jump between those is difficult*.*’* (Dr A, 52 years, woman, GP partner)

Therefore, when healthcare professionals were expecting thrush to be acute, recurrence often fell outside the boundaries of what existing systems were designed to diagnose and treat.

#### Examinations

Patients and healthcare professionals explained that physical examinations could aid recurrent thrush diagnoses, sometimes helping to visualise discomfort, and other times to rule out other conditions:

*‘I did believe it was necessary for the nurse to see it because she saw the gravity of it.’* (Hannah, 31 years, woman, patient)

However, not everyone was examined, and sometimes recurrent thrush was diagnosed based on symptom description alone:

*‘Quite quickly I was saying, “I know I've got thrush”, and no one looked, I mean barely any physical exams, just a conversation with me describing my symptoms, and then prescribing me* [laughs] *the same stuff. I think I asked a couple of times for people to look, and so they did; that’s all*.’ (Anna, 34 years, woman, patient)

Some patients were unsure whether an examination was necessary for recurrent thrush and if it was expected or appropriate to ask for one:

*‘I felt like it wouldn’t be appropriate for me to ask for it because I don’t know what or if examinations are supposed to be done. I would prefer that someone examines me, but if that’s not something that people usually do when they have thrush, then that’s all right, then it’s just a preconception that I have that whenever I go to the doctor, they’re supposed to examine me to see what’s wrong*.’ (Aditi, 22 years, woman, patient)

Healthcare professionals highlighted the importance of vulval and vaginal examinations and consistently offering these to patients with suspected recurent thrush:

*‘I would almost always offer an exam, particularly if it was ongoing, particularly if it hadn’t responded to treatment, particularly if we’re really trying to pin down what it is and inform ongoing treatment*.*’* (Dr A, 52 years, woman, GP partner)

*‘I think the not looking* [at the vulva and vagina] *I would see as dismissive. And why are* [healthcare professionals] *not looking. I think it’s just wrong*.*’* (Dr Q, 53 years, woman, GP)

#### Access to different types of testing

Testing availability varied across services. General practices often did not have access to the same testing as sexual health centres, such as investigation into fungal species and sensitivities:

*‘There are far greater challenges in primary care where they don’t have microscopy services to label something as a recurrent problem*.*’* (Dr D, 38 years, man, sexual health consultant)

*‘Our* Candida *testing in primary care isn’t brilliant* […] *What we get back from microbiology are samples saying “insufficient”, or broadly saying “Normal vaginal bacteria”.’* (Dr F, 35 years, woman, GP)

Therefore, what tests were available, which results were possible, and which services could access them varied by setting, practice, and location.

### Navigating services and permeability

#### Expectations around repeat testing

Clinical guidelines require two tests showing the presence of a *Candida* species to diagnose recurrent thrush, meaning multiple appointments were required and an anticipated time delay. However, these timelines were not always understood or adequately explained to patients who held differing expectations.

Some patients found it difficult to access multiple appointments and build up enough positive swabs to show recurrent thrush. Zoya explained the prolonged process of accessing repeat testing:

*‘It was six to nine months and then I felt like I was getting somewhere. They were like, “OK, recurrent thrush.”’* (Zoya, 33 years, woman, patient)

Zoya goes on to explain that when she became pregnant she did not experience thrush during this time and stopped seeking care. When her thrush symptoms returned, she re-visited the GP, but was disappointed to have to restart the process of documenting recurrence:

*‘I said, “Look at my old records, how many times I’d been, look at my swabs.” I had to do more swabs again to get them to diagnose me* [with] *recurrent thrush. I needed a certain number of swabs or episodes*.’ (Zoya, 33 years, woman, patient)

How many swabs were required, who could collect and document these, and in what timeframe were rarely made explicit to patients.

#### Patients self-treating before testing

Some patients found it difficult to make repeat appointments with healthcare professionals while symptomatic, because of fluctuating flare-ups and limited appointment availability. Others said it felt impossible to endure discomfort while awaiting an appointment and would self-treat with antifungal medication, which would then affect test accuracy:

*‘You think, “I know having this recorded on a system would be hugely beneficial because* [the GP] *can record how many episodes I’ve had”, but the symptoms become too intolerable*.*’* (Emma, 41 years, woman, patient)

Clinicians recognised the challenges associated with gathering accurate swab results:

*‘There can be challenges when they couldn’t get the appointment in time and then it’s gone and we can’t prove it* […] *There can be problems when* [the patient] *comes, but they were so desperate, they tried to treat it the night before and then the swab comes back negative and you don’t know — it could’ve been thrush or not*.’ (Dr Q, 53 years, woman, GP)

To address these challenges, some healthcare professionals and patients explored the options for self-testing, whether through a healthcare professional or commercial option.

### Accessing services

#### Clinician-initiated at-home swabs

If a patient presented without active symptoms, primary care professionals spoke about offering swabs for patients to take at home and return them to the clinic in the context of an agreed plan.

Self-swabbing meant patients could test when they were most symptomatic and before self-treating. Patients often found having a self-swab at home helpful:

*‘*[I was told] *when I had symptoms, to swab myself, which is easy, and then hand it back into the clinic to test it*.*’* (Emily, 32 years, woman, patient)

However, not all healthcare settings offered this option, and patients reported confusion about where and when self-testing was available. Some physicians were hesitant about offering these options:

*‘I think self-swabs can be tricky in terms of are they complying and handing it back*.*’* (Ms I, 35 years, woman, physician associate)

#### Commercial self-testing kits

Some patients investigated a diagnosis independent from (or in addition to) healthcare professionals. Self-testing kits, such as pH test strips and vaginal microbiome test kits, were available at pharmacies, online, and in some supermarkets. Patients sometimes interpreted these results themselves whereas others presented them to clinicians.

Healthcare professionals acknowledged that current at-home tests available to consumers were not necessarily reliable, accurate, or informative:

*‘I know this litmus paper* [one form of commercial self-testing kit] *for patients, I don’t think that’s good enough.’* (Dr J, 59 years, woman, GUM consultant)

*‘*[The pH tests] *were temperamental. One week I’d have evidence for what I was feeling. The other week it would be the other way. They weren’t reliable, but at the time they gave me the reassurance I needed that it wasn’t in my head*.*’* (Ayesha, 25 years, woman, patient)

If patients bought their own self-testing kits it was unlikely that the thrush episodes were being recorded, making pattern recognition more difficult:

*‘A GP will recommend, “just get a self-testing kit”, but what do you do with the results? I appreciate it’s a convenient option, but it’s not being recorded in your medical notes*.’ (Emma, 41 years, woman, patient)

Some patients brought in test results from commercial at-home vaginal microbiome test kits, but healthcare professionals reported being unable to action the results as evidence for how such tests can inform care is lacking.

### Adjudication

#### Belief in reliability and accuracy of test results

Interpreting test results for recurrent thrush (whether administered from healthcare professionals, or accessed at home) was not necessarily straightforward, and needed to be understood within a larger clinical context:

*‘I wouldn’t rely on a swab, it’s not the sort of thing that everything hinges on. It would be part of a jigsaw puzzle.*’ (Dr P, 44 years, woman, GP partner)

Asymptomatic carriage of *Candida* species could be present on swabs, but not necessarily be causing vulvovaginal irritation:

*‘If you look at one hundred women or people with a vagina, ten to fifteen will have* Candida *if you swab them, but they’re not having any symptoms*.*’* (Dr J, 59 years, woman, GUM consultant)

Patients also worried that, if the cause of symptoms was not thrush, the swab would not necessarily reveal what else it could be:

*‘If they send* [a swab] *off to the lab and it’s thrush — they know it’s thrush, but if it wasn’t thrush and I was getting similar symptoms, it could be something else*.’ (Leah, 26 years, woman, patient)

#### Separating recurrent from persistent cases of thrush

Patients and healthcare professionals noted variation in duration and frequency within recurrent thrush cases and recommended seperating out these instances. Recurrent thrush was presented as having distinct periods of symptoms and treatment-responsive relief:

*‘A textbook case* [of recurrent thrush] *would be “I use the treatment, it goes away, and I feel all right for two weeks and then it comes back*.”’ (Dr A, 52 years, woman, GP partner)

On the other hand, persistent or chronic thrush was described as enduring or lingering:

*‘*[Persistent thrush] *runs in the background, maybe it gets a bit better for a few days then has another flare-up.’* (Dr D, 38 years, man, SH consultant)

Clinicians suggested that, in persistent or chronic cases of thrush, the culprit was typically a fluconazole-resistant strain of *Candida*, not thrush, or not solely thrush. Treating with a long-term fluconazole course and re-testing was suggested as a way to help identify whether symptoms were because of thrush or a differential diagnosis:

*‘What we would try to do is cure, as in get rid of the* Candida*. Then take a swab. If they still have symptoms, well there was no Candida. So, let’s leave the Candida alone, that’s not the cause of symptoms*.*’* (Dr O, 47 years, woman, SH consultant)

#### Recognising not all itch is thrush

Patients often reported symptoms that were vague, non-specific, or undifferentiated. Sometimes these symptoms were labelled as recurrent thrush when another condition was present that could be resolved with different treatment:

*‘It’s just a perception that itching is thrush, but it might or might not be. Do not just say: “You’ve got thrush.” You could be two years down the line and it was never thrush, it was lichen sclerosus*.*’* (Dr Q, 53 years, woman, GP)

Patients expressed uncertainty about whether recurrent thrush was their accurate or only diagnosis. Some were later diagnosed with other conditions, such as lichen sclerosus, vulvodynia, dermatitis, or vaginismus. Misattributing symptoms to thrush could delay appropriate diagnosis, lead to ineffective treatment, and cause further complications.

### Offers and resistance

#### Finding the ‘right’ language and labels

Patients and healthcare professionals held differing beliefs about the ‘right’ words to label the experience:

*‘They never gave me an official diagnosis* […] *It was never a label where they said, “You've got recurring thrush.” It was more, “We can see you’ve got a history of repeated episodes of thrush.’”* (Jody, 26 years, woman, patient)

This example highlights the different meanings people placed on labels. Some healthcare professionals expressed reluctance to use the term ‘recurrent thrush’, unless the patient did first:

*‘I tend to mirror the language they’re using, so unless they say, “I have recurrent thrush”, I might say, “Look, you’re somebody who’s very prone to thrush.”’* (Dr B, 24 years, woman, foundation year doctor)

*‘I might describe recurrent as: “It keeps coming back”, but I don’t know if I’ve ever said, “You have recurrent thrush.” It seems quite final, like they’re always going to have recurrent thrush. I want to sound more hopeful*.*’* (Dr H, 32 years, woman, GP)

However, patients described uneasiness after hearing the frequent remark that this condition was *‘*just *thrush’*. Kayla (42, woman, patient) said clinicians *‘don’t seem interested in* [recurrent thrush]*, they say, “Oh, it’s just thrush.”’* Healthcare professionals’ comments describing thrush as *‘normal’*, *‘common’*, or *‘*just *thrush’* could be intended to reassure, but often were perceived as unhelpful or dismissive. The term ‘just’ not only labels recurrent thrush as trivial, but also lumps all types of thrush together regardless of frequency and endurance.

Offering a label could help patients better understand their condition, plan a management approach, and validate their experience:

*‘I just felt like I finally knew what the name was for what I was feeling*.*’* (Aditi, 22 years, woman, patient)

*‘Sometimes the formality of a phrase, rather than “thrush that keeps coming back”, feels like a positive medical label they can use: “It’s not just thrush, it’s recurrent thrush.”’* (Dr X, 40 years, woman, GP community gynaecology)

Labels of recurrent thrush not only offered validation, but also enabled patients to receive long-term antifungal medication, build ongoing healthcare relationships, and develop ongoing management approaches.

#### Bringing the guidelines into consultations

Healthcare professionals discussed how they explained to patients their reasoning around reaching a recurrent thrush diagnosis (or not). To establish authenticity, transparency, and trustworthiness, some healthcare professionals showed patients the clinical guidelines and explained their thought process:

*‘I usually bring the guidelines up and say, “Would you say you fall into this category? And if so, what are we going to do?”’* (Dr A, 52 years, woman, GP partner)

Some patients had consulted national guidelines during their own research into recurrent thrush, and this helped them better understand the process and label of recurrent thrush:

*‘I looked on the NICE* [National Institute for Health and Care Excellence] *guidelines. It was like, “right, OK, I can say that to them now” and I went to the GP*.*’* (Sarah, 35 years, woman, patient)

By bringing up the guidelines, healthcare professionals and patients were able to work together to manage expectations and create a plan to build a diagnostic picture.

## Discussion

### Summary

This study explored how recurrent vulvovaginal thrush is recognised and diagnosed in primary care, from the perspectives of patients and healthcare professionals. Our findings demonstrate that the label of recurrent thrush may, for a variety of reasons, not be easily established or applied in healthcare settings. Numerous steps were needed for recurrent thrush to be investigated, diagnosed, and communicated, some of which required action from patients and others from healthcare professionals. Diagnosis was recognised as a process that involved identifying patterns of recurrence, documenting this recurrence, accessing testing, interpreting test results, and forming a diagnosis as a label that enabled management pathways.

### Strengths and limitations

This study is the first to combine the views of healthcare professionals and patients to explore recurrent thrush diagnosis in England. Our sample included patients and healthcare professionals from a range of locations and with diverse backgrounds and identities. By combining the views of patients and healthcare professionals, this paper highlights opportunities for working together to improve diagnostic experiences.

Some limitations include patients presenting retrospective accounts from the perspective of now knowing or thinking they were living with recurrent thrush. Therefore, past experiences with vague symptoms may be re-interpreted or retrofitted through a lens of recurrent thrush.

Healthcare professionals may have felt the need to review or reference guidelines, therefore they were prompted to share examples rather than hypothetical responses. Our findings have been considered in this context and presented as accounts providing insight into these perspectives.

This study included healthcare professionals working across different health settings (GPs, nurses, sexual health consultants, community gynaecology, and so on), but not pharmacists. As recurrent thrush often presents to clinicians when over-the-counter options are insufficient, we wanted to examine this area of diagnosis. With increasing momentum towards pharmacy-first care for vulvovaginal symptoms, our findings caution that this pathway (with no examinations or swabs) might have unintended consequences of working against a diagnosis of recurrent thrush and similar conditions. Future research should investigate how these trends and the rise of self-diagnosis technologies shape diagnosis and care experiences.

### Comparison with existing literature

Our findings support past work that general practice plays a pivotal role in early recognition of vulvovaginal conditions, but that complex challenges exist.^27^ Clinical guidelines in the UK published by NICE and the British Association for Sexual Health and HIV (BASHH) outline the process to diagnosis. However, existing studies report inconsistencies in healthcare professional approaches to recurrent thrush and adherence to diagnostic guidelines.^9,10^ Although our study supports that diagnosing recurrent thrush can be difficult, it is not simply because of a lack of knowledge or guidance. Instead, our findings highlight areas where clinicians were largely following national guidelines, but differing expectations, perceptions, or understanding from patients led to perceived missed opportunities.

This paper provides insight into previously reported problems with diagnosis, and the misattribution of other vulval conditions being mislabelled as recurrent thrush, such as lichen sclerosus and vulvodynia.^11,14,15,28^ This includes diagnosis being made on symptom description alone without physical examinations, and limitations with current diagnostic tools and tests. Our study supports calls to address these limitations.^16,27^

The Candidacy Framework outlines how people identify a health problem, seek care, and negotiate outcomes, but overlooks aspects of recurrent conditions.^24^ Updates adding recursivity (returning to earlier stages) help explain recurrent thrush, where patients repeatedly cycle through stages.^26^ However, for recurrent conditions, recursivity is not optional, but central as candidacy hinges on when a symptom is recognised as a pattern, and whose timeline defines ‘recurrence’. Our study found mismatches between patient experiences and institutional abilities to capture recurrence. The stages of candidacy could apply (although not always equally) across patient and healthcare professional accounts, with many ‘stages’ occurring simultaneously or in different orders.

**Figure 1.**
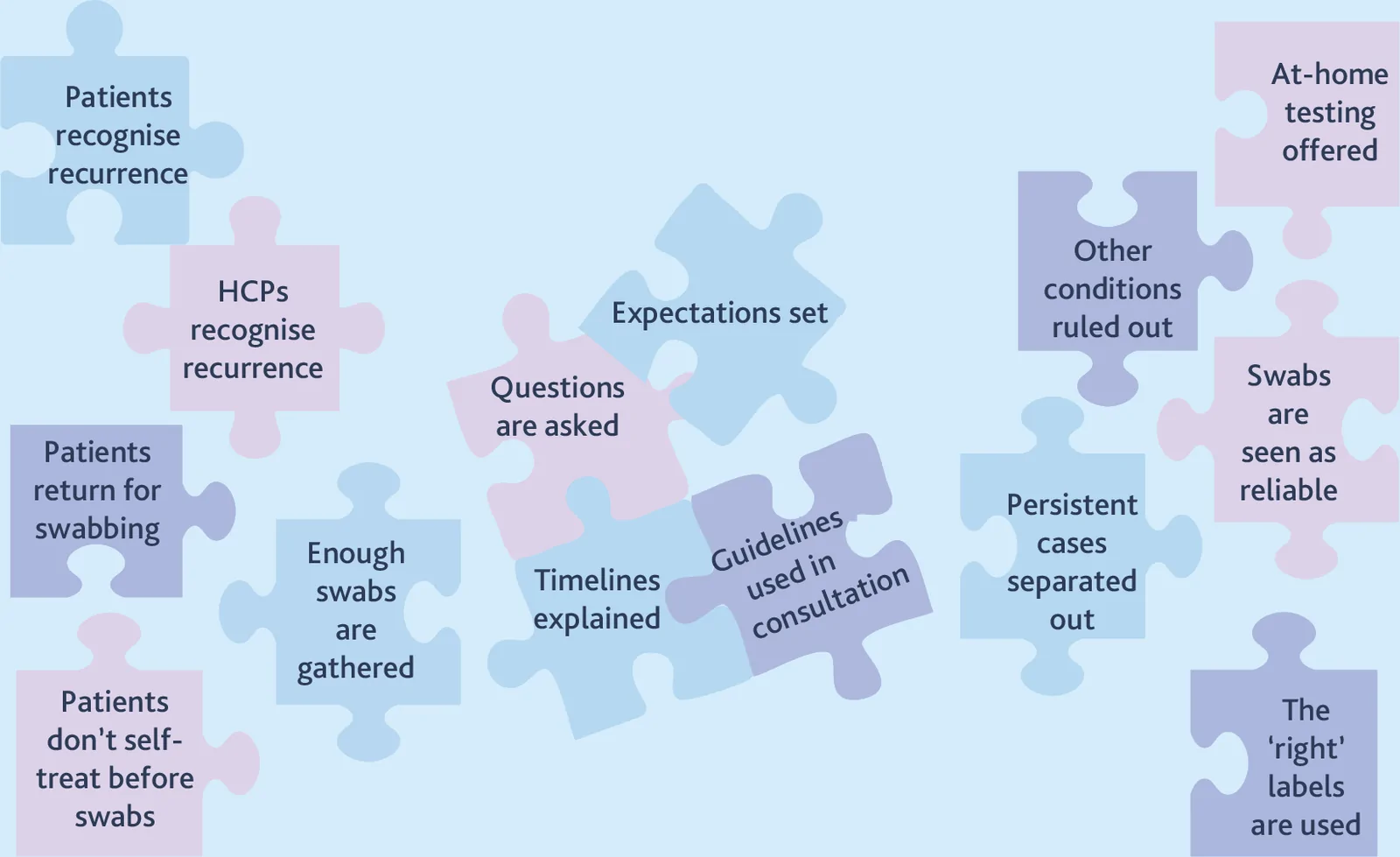
The jigsaw puzzle of diagnosing possible recurrent vulvovaginal thrush. HCP = healthcare professional.

As one healthcare professional described, recurrent thrush presented a ‘jigsaw puzzle’ for diagnosis, with multiple pieces picked up and considered before attempting to click them all together. Figure 1 outlines the multiple pieces of the puzzle that patients and/or healthcare professionals were holding, comparing, and trying to make fit.

### Implications for research and practice

This article identifies a need to enhance the recognition and diagnosis of recurrent thrush in primary care. Acute cases of thrush are defined in guidelines as the ‘first’ isolated presence of thrush; however, there are likely distinctions between when thrush is first experienced by an individual, and when they present to a healthcare professional. Further, although the guidelines recommend two positive swabs for diagnosis, they do not capture challenges with obtaining reliable swabs, interpreting results, addressing at-home test kits, or considering the variable definitions and language surrounding this condition.

Within this context, we provide some recommendations within primary care (Box 1).

Box 1. **Recommendations to improve diagnosis of recurrent vulvovaginal thrush**

- **Prompt patients about recurrent and persistent symptoms**: ask how many episodes of suspected thrush they have experienced in a year, ask if symptoms resolve between episodes
- **Ask about self-treatment before appointments**: explain how self-treating may influence test results, encourage patients to not self-treat before testing
- **Offer patient-initiated self-swabs**: support pathways for self-swabbing, and explain that patients should swab at home when symptoms are active (before treatment) and then return the swabs to the clinic to be tested
- **Explain diagnostic criteria**: be explicit about requiring two swabs confirming thrush within a 12-month period
- **Acknowledge the limitations of current tests**: explain that swabs may detect thrush that is not the cause of problems
- **Offer examinations to all patients with recurrent vulvovaginal symptoms**: ensure that all possible differential diagnoses are considered
- **Educate patients on other conditions that cause vulval itch**: help clinicians and patients work together to find an underlying cause

Further research could explore:

- improved recognition, diagnostic tools, and distribution across GP services, sexual health, and pharmacies;
- the diagnostic accuracy, value, and interpretation of commercial self-testing options; and
- the role of prophylactic antifungal treatment in helping diagnose and manage recurrence.

This study highlights how recurrent vulvovaginal thrush diagnosis is a negotiated, iterative process shaped by patient experience, professional judgement, and access to diagnostic tools. By exploring patient experiences and healthcare professional perspectives, we provide insight into previously reported diagnostic delays. Our findings highlight areas where clinicians were largely following national guidelines, but differing expectations, perceptions, or understanding from patients led to dissatisfaction and perceived missed opportunities in diagnostic pathways. Diagnosing recurrent thrush was not solely a matter of locating biological proof of a fungal organism, seeing symptoms, or hearing patient stories, but a careful choreography of making a condition legible and actionable. The findings highlight opportunites for improved communication, better diagnostic tests, and consistent language to improve the identification and investigation of possible recurrent vulvovaginal thrush in primary care.

## References

[1] National Institute for Health and Care Excellence (NICE) (2023). Candidiasis – female genital. https://cks.nice.org.uk/topics/candida-female-genital/.

[2] Saxon C, Edwards A, Rautemaa-Richardson R (2020). British Association for Sexual Health and HIV national guideline for the management of vulvovaginal candidiasis (2019). Int J STD AIDS.

[3] Chapple A, Hassell K, Nicolson M, Cantrill J (2000). ‘You don’t really feel you can function normally’: women’s perceptions and personal management of vaginal thrush. J Reprod Infant Psychol.

[4] Erfaninejad M, Salahshouri A, Amirrajab N (2022). Barriers and facilitators of adherence to treatment among women with vulvovaginal candidiasis: a qualitative study. Eur J Med Res.

[5] Karasz A, Anderson M (2003). The vaginitis monologues: women’s experiences of vaginal complaints in a primary care setting. Soc Sci Med.

[6] Morgan M, McInerney F, Rumbold J, Liamputtong P (2009). Drawing the experience of chronic vaginal thrush and complementary and alternative medicine. Int J Soc Res Methodol.

[7] Theroux R (2002). Bypassing the middleman: a grounded theory of women’s self-care for vaginal symptoms. Health Care Women Int.

[8] Ford T, Talbot A, Hayward G (2024). Managing recurrent vulvovaginal thrush from patient and healthcare professional perspectives: a systematic review and thematic synthesis. Patient Educ Couns.

[9] Adolfsson A, Hagander A, Mahjoubipour F, Larsson P-G (2017). How vaginal infections impact women’s everyday life — women’s lived experiences of bacterial vaginosis and recurrent vulvovaginal candidiasis. ASM.

[10] Bradfield Strydom M, Walpola RL, McMillan S (2022). Lived experience of medical management in recurrent vulvovaginal candidiasis: a qualitative study of an uncertain journey. BMC Womens Health.

[11] Leusink P, Teunissen D, Lucassen PL (2018). Facilitators and barriers in the diagnostic process of vulvovaginal complaints (vulvodynia) in general practice: a qualitative study. Eur J Gen Pract.

[12] Neal CM, Martens MG (2022). Clinical challenges in diagnosis and treatment of recurrent vulvovaginal candidiasis. SAGE Open Med.

[13] Nunns D, Murphy R (2012). Assessment and management of vulval pain. BMJ.

[14] Farage MA, Miller KW, Summers PR (2010). Chronic pain of the vulva without dermatologic manifestations: distinguishing among a spectrum of clinical disorders. Clin Med Insights Womens Health.

[15] Arnold S, Fernando S, Rees S (2022). Living with vulval lichen sclerosus: a qualitative interview study. Br J Dermatol.

[16] Ascott A, Chinthapalli S, Gibbon K (2017). Unifying clinical care between specialties: a model for genital disease. J R Soc Med.

[17] Dixon S, McNiven A, Talbot A, Hinton L (2021). Navigating possible endometriosis in primary care: a qualitative study of GP perspectives. Br J Gen Pract.

[18] Ford T, Tonkin-Crine S, Hayward G (2026). Accumulative experiences of recurrent vulvovaginal thrush: accumulative experiences from patient and clinician perspectives. i.

[19] Meyer T, Xyländer M, Lucius-Hoene G, Holmberg C, Meyer T (2018). Illness narratives in practice.

[20] Grob R, Schlesinger M, Parker AM (2016). Breaking narrative ground: innovative methods for rigorously eliciting and assessing patient narratives. Health Serv Res.

[21] Ziebland S, McPherson A (2006). Making sense of qualitative data analysis: an introduction with illustrations from DIPEx (personal experiences of health and illness). Med Educ.

[22] Spalding NJ, Phillips T (2007). Exploring the use of vignettes: from validity to trustworthiness. Qual Health Res.

[23] Hughes R, Huby M (2002). The application of vignettes in social and nursing research. J Adv Nurs.

[24] Dixon-Woods M, Cavers D, Agarwal S (2006). Conducting a critical interpretive synthesis of the literature on access to healthcare by vulnerable groups. BMC Med Res Methodol.

[25] Sinnott C, Ansari A, Price E (2024). Understanding access to general practice through the lens of candidacy: a critical review of the literature. Br J Gen Pract.

[26] Koehn S, Jones CA, Barber C (2024). Candidacy 2.0 (CC) — an enhanced theory of access to healthcare for chronic conditions: lessons from a critical interpretive synthesis on access to rheumatoid arthritis care. BMC Health Serv Res.

[27] Rees S, Owen C, Baumhauer C, Hillman S (2023). Vulval lichen sclerosus in primary care: thinking beyond thrush and genitourinary symptoms of the menopause. Br J Gen Pract.

[28] Crew A, Leatherland R, Clarke L (2025). Barriers to diagnosing and treating vulval lichen sclerosus: a survey study. Br J Gen Pract.

